# The role of hydroxyurea in modulating miRNA expression in sickle cell disease: molecular mechanisms and therapeutic implications

**DOI:** 10.1007/s00277-026-06969-3

**Published:** 2026-04-22

**Authors:** Faezeh Mirzaee, Atefeh Khamoushi, Roghayeh Dolati, Amirhosein Abbasi

**Affiliations:** 1 Student Research Committee, Jiroft University of Medical Sciences, Jiroft, Iran; 2 Antigen Antibody Engineering Department, National Institute of Genetic Engineering and Biotechnology (NIGEB), Tehran, Iran; 3 Department of Biophysics, Faculty of Bioscience, Tarbiat Modares University, Tehran, Iran

**Keywords:** Sickle Cell Disease (SCD), Hydroxy urea, miRNA regulation, Hemoglobin S (HbS), Oxidative stress

## Abstract

Sickle Cell Disease (SCD) is a common autosomal recessive hemoglobinopathy caused by a point mutation in the β-globin gene, leading to aberrant production of Hemoglobin S (HbS). The pathophysiological process initiated by this mutation triggers a cascade of symptoms, including erythrocyte sickling, vasococcus occlusions, chronic hemolysis, and widespread inflammatory reactions, culminating in a multitude of clinical complications. Unfortunately, in resource-poor regions, the increasing incidence of SCD outstrips the capabilities of public healthcare systems, thereby creating a dire and urgent requirement for readily available and optimized therapies. Hydroxyurea (HU) has proven itself as a definitive treatment that promotes fetal hemoglobin production, prevents clinical complications, and thereby provides a promising, cost-effective alternative to chronic blood transfusions. Recent findings indicate that microRNAs (miRNAs) play a pivotal role in regulating hematopoiesis and globin switching, thereby making them fundamental mediators and targets of HU therapy. In this review, we explore beyond the traditional frontiers of HU therapy by critically evaluating its rationale as a two-way modifier of the miRNA-scope, which affects crosstalk within a ceRNA network, promotes upregulation of desirable miRNAs, and also triggers HU-induced adaptive miRs that can potentially destabilize therapeutic responses or even induce adverse reactions. More importantly, this review proposes a new, comprehensive conceptual framework to overcome and explore the constraints of monotherapy. In a nimble review of prior findings, we clarify and establish which specific alterations of HU-induced miRs would form a rational basis and hypotensive stimulus of mimicking therapy and thereby differentiate which specific alterations of upregulated or downregulated miRs should form a systemic basis of antagomiring therapy and thus lay out a specific and comprehensive roadmap of combinatorial medicine and therapy in managing SCD.

## Introduction

Sickle Cell Disease (SCD) is a genetic blood disorder characterized by the production of abnormal hemoglobin molecules that cause red blood cells to become crescent- or sickle-shaped. People with SCD may experience recurring pain, fatigue, anemia, increased susceptibility to infections and arterial occlusion, and acute chest syndrome, which may lead to premature death [1]. SCD has serious economic and social impacts and can cause serious damage to health and social systems [1].

Treatment options for SCD focus on the management of symptoms and prevention of complications, including pain management using analgesics, hydration, and blood transfusion. Sickle cell anemia (SCA) management includes both acute and chronic complications, including pain management strategies for Vaso-Occlusive Crisis (VOC) and transfusion management to prevent stroke. Currently, HU is the only approved first-line drug by the US Food and Drug Administration (FDA) and European regulatory authorities [2]. This is the only ideal and cost-effective drug with clinical efficacy and good oral bioavailability for treating patients with SCD. HU is a potent inducer of Hemoglobin F (HbF) and a myelocytic suppressor, effective in improving the hematologic status of sickle cell patients by stimulating the production of young red blood cells with high hemoglobin F and low propensity to polymerize, thereby decreasing sickling and increasing hematocrit [3, 4].

Hydroxyurea inhibits ribonucleotide reductase, inducing controlled myelosuppression and reducing neutrophil, platelet, and reticulocyte counts [3]. This is not an adverse event but rather a mechanism of action. Neutrophils are involved in vaso-occlusion through their adhesion to the vascular endothelium and interaction with sickled erythrocytes. Reducing neutrophil numbers through myelosuppression can decrease adhesion and reduce the number of vaso-occlusive crises in SCD patients. The MSH trial showed that lower neutrophil counts were independently associated with reduced crises. Therefore, specific myelosuppression is a marker of appropriate dosing and, together with HbF induction, can lead to improved clinical responses in SCD patients [4]. Long-term HU therapy results in sustained hematologic benefits, fewer acute chest syndrome events, improved growth, and possibly preserved organ function. These ameliorating effects improve patient outcomes, including reduced rates of occlusive crises, blood transfusions, and hospitalizations, as well as improved overall survival [5].

MicroRNAs (miRNAs) are widespread and influential molecular regulators that modulate the expression of numerous protein-coding genes by suppressing mRNA translation. Studies have shown that miRNAs play a role in cell regulation and differentiation, including hematopoiesis and hemoglobin replacement [6, 7]. In this article, we review the relationship between HU and microRNAs in the cell cycle to gain a better understanding of its mechanism and to create a more effective treatment for patients.

## miRNAs increased by HU treatment

### MicroRNA-29

**Fig. 1:**
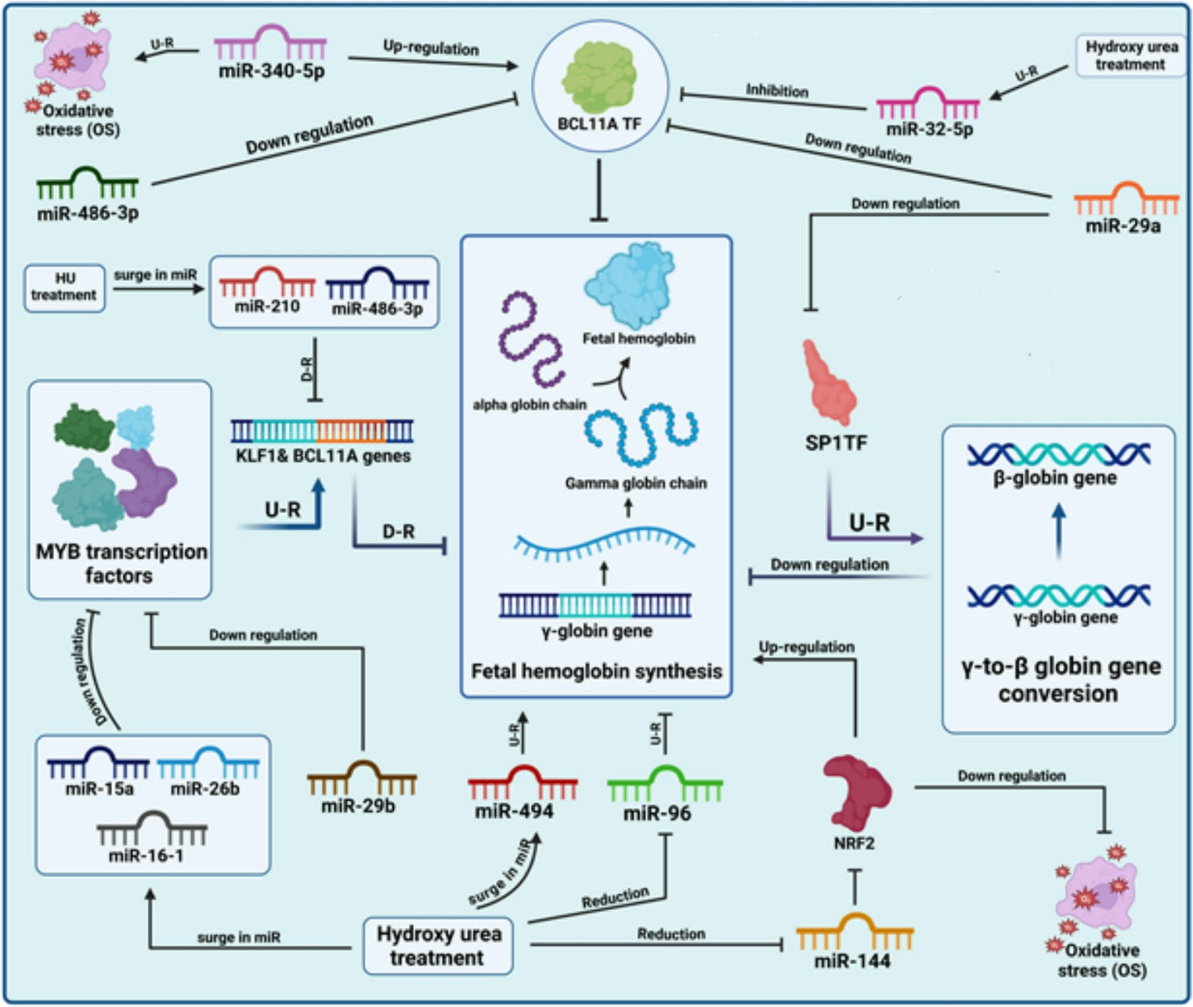
The mechanism of the interactions between HU and miRNA (mir-15a [17], mir-26b [17], mir-16-1 [10], mir-29b [10], mir-494 [10], mir-96 [10], mir-144 [38], mir-210 [10], mir-486-3p [24], mir-29a [10], mir-340-5p [7], mir-32-5p [40]) in fetal hemoglobin synthesis, y-to-β globin gene conversion and ultimately the treatment of SCA, U-R and D-R refers to upregulation and downregulation, respectively

miR-29a plays a role in controlling inflammation, fat processing, and bone development and might help in treating SCA by controlling BCL11A and SP1 [7–9] Fig. 1.

BCL11A is a master transcriptional regulator of γ-globin genes, and its downregulation is a known mechanism for stimulating fetal hemoglobin (HbF) synthesis. Kargutkar et al. in 2023 have investigated the total transcriptome of CD71+ (transferrin receptor 1) erythroid cells from SCD patients versus normal individuals after HU therapy, and the results showed the increased levels of miR-29a, which targets BCL11A mRNA, leading to decreased BCL11A expression and subsequent decreased γ-globin gene expression [10]. In addition, miR-29a, miR-23a, and miR-27a can target and inhibit the expression of the SP1 transcription factor important in the γ-to-β globin gene conversion [11].

Experimental studies have shown that miR-29b can target and down-regulate the expression of the MYB transcription factors, which is a major regulator of γ-globin genes, and, like miRNA-29a, miRNA-29b also regulates BCL11A and *KLF1* and may be helpful in clinical SCA [12].

### MicroRNA-144

MiR-144 is highly expressed in individuals with SCD compared to healthy individuals. Increased miR-144 expression is associated with more severe anemia in individuals with SCD [10]. MiR-144 modulates NRF2 expression, an essential transcription factor that regulates the cellular response to oxidative stress. High expression of miR-144 leads to low expression of NRF2, which decreases the ability to resist oxidative stress and replenish glutathione in sickle-shaped red blood cells [13] Fig. 1.

NRF2 is a strong inducer of HbF production, which may interfere with the polymerization of sickle hemoglobin (HbS), a major pathogenic mechanism in SCA. The chronic destruction of red blood cells and inflammation in SCA leads to high oxidative stress. Reduced NRF2 function further aggravates oxidative stress, inflammation, and tissue damage in SCA [14]. This reduced tolerance to oxidative stress contributes to the more severe anemia features observed in SCD subjects with high miR-144 levels. Building on these observations, suppression of miR-144 expression may be a potential therapeutic approach to increase tolerance to oxidative stress and reduce the clinical severity of SCA. In this context, a microarray-based miRNA expression profiling assay conducted by Biaoru Li et al. in 2019 demonstrated an eightfold upregulation of miR-144-3p (miR-144) and miR-144-5p in the reticulocytes of the low-HbF group compared with the high-HbF group. Furthermore, subsequent functional studies in normal and sickle erythroid progenitors demonstrated that miR-144 silences NRF2 and simultaneously suppresses γ-globin transcription. Notably, treatment with the miR-144 antagomir reversed these silencing effects in a dose-dependent manner [15].

### MicroRNA-210

The rise in miR-210 expression is largely associated with the increase in fetal hemoglobin (HbF) levels and the protection of cells from death due to a lack of oxygen [16].

In a 2023 study by Kargutkar et al., the association between miR-210 and increased γ-globin gene expression in CD71 + cells was demonstrated, achieved by inhibiting the activity of negative regulators such as BCL11A and KLF-1. Furthermore, miR-210 may increase HbF levels by inhibiting BCL11A [10]. Excessive miR-210, along with miR-494 and miR-130, protects cells from oxygen deficiency-triggered cell death in sickle cell patients receiving HU therapy. This protective process is likely due to miR-210’s ability to regulate genes involved in red blood cell development and cell survival under low-oxygen conditions.

 [7, 10] Moreover, miR-210 plays a role in red blood cell maturation and in the induction of HbF in patients with SCD undergoing HU therapy. Its presence is positively linked to HbF levels in response to HU treatment [10] Fig. 1.

### MicroRNA-16-1

the sudden increase in miR-16-1 expression is also demonstrated in CD71 + erythrocytes of SCD patients and normal individuals after HU treatment [10]. As an element of post-transcriptional regulation, miR-16-1 is part of the miR-15a/16 − 1 cluster, which modulates the expression of the MYB transcription factor [17–20]. Heightening miR-16-1 levels correlates with reduced MYB expression, relieving the inhibition of γ-globin gene expression and ultimately boosting HbF production [10, 17] Lower levels of MYB lead to slower cell cycle advancement and expedited differentiation in erythroid cells, which can stimulate the production of more “F-cells,” predominantly generating HbF prior to the conversion to adult hemoglobin [8, 16] (Fig. 1).

### MicroRNA-320

MiR-320 is an important physiologic regulator of CD71, controlling red blood cell maturation and survival, as well as iron homeostasis. In SCA, abnormally low miR-320 levels increase CD71 expression, leading to erythropoietic defects and poor cell survival [19]. Correction of miR-320 expression augmentation will reverse these processes, restore iron homeostasis, and maintain normal erythropoiesis [10, 21] The increased expression of miR-320 by HU in CD71 + erythroid cells is associated with elevated HbF production and improved red blood cell maturation. In addition, the increased levels of miR-320, reduces the expression of CD71 which are dysregulated in most cases of SCA [12, 22, 23].

### MicroRNA-486-3p

miR-486-3p regulates fetal hemoglobin (HbF) levels by targeting the BCL11A gene. These effects have been demonstrated in in vitro studies using human CD34 + erythroid progenitors [24] Fig. 1.

Clinical studies in patients with SCD and β-thalassemia have shown that HU responders have higher miR-486-3p levels than non-responders, which correlate with increased HbF levels.

 [25]. Mechanistic insights from Kargutkar et al. indicated that HU modulates miRNA networks, including miR-486-3p, to suppress BCL11A [10]. Together, these data suggest that miR-486-3p mediates part of HU’s therapeutic effect; however, further research is needed to fully understand its potential for targeted HbF induction in SCD [26].

### MicroRNA-494

Moreover, miR-494 acts as an HU-induced promoter of fetal hemoglobin production in SCD. In CD71 + erythroid cells from patients with SCD, the expression of miR-494, miR-130, and miR-210 was increased under hypoxic conditions following HU treatment. Increased miR-494 expression was also associated with elevated HbF levels in these patients following HU treatment [10].

### MicroRNA-130b

**Fig. 2:**
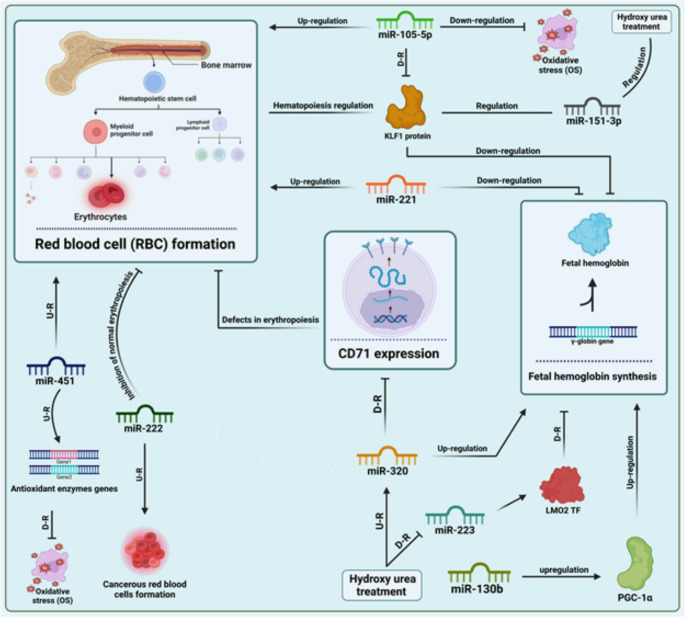
The mechanism of the interactions between HU and miRNAs (mir-451 [31], mir-222 [59], mir-130b [28], mir-223 [10], mir-320 [23], mir-221 [59], mir-105-5p [7], mir-151-3p [41]) in red blood cell formation, fetal hemoglobin synthesis and ultimately the treatment of SCA, U-R and D-R refers to upregulation and downregulation, respectively

miR-130b inhibits cell growth, encourages apoptosis, and inhibits the PI3K/Akt pathway [27]. Low oxygen levels and increased expression of miR-494, miR-130, and miR-210 in CD71 + erythroid cells of patients with SCD after HU protect cells against low-oxygen-induced apoptosis [10, 25]. Peroxisome proliferator-activated receptor gamma-activator 1α (PGC-1α) is a master regulator of mitochondrial biogenesis and function. Sun et al. (2022) reported that increasing PGC-1α expression by infection with a lentivirus expressing PGC-1α or by a small molecule PGC-1α agonist called ZLN005 in human CD34 + early erythroid progenitor cells induced fetal γ-globin mRNA and protein expression as well as the percentage of HbF-positive cells (F cells) without significantly affecting cell proliferation and differentiation. The combination of PGC-1α and HU stimulators may provide an effective therapeutic strategy. MiR-130b-3p significantly affects PGC-1α expression and could be useful for therapy [28, 29] (Fig. 2).

### MicroRNA-451

Elevated miR-451 levels have important effects on SCA by affecting red blood cell differentiation and hemoglobin production [30]. Shokri et al. in 2019 have indicated that raised miR-451 levels encourage red blood cell differentiation and the development of mouse embryonic stem cells (mESCs) without the requirement for stimulating cytokines. This increase also results in heightened expression of various globin chains, such as α-globin, β-globin, and embryonic globins (ζ and ε), which typically increase in the later stages of red blood cell production, suggesting a role in the progression of red blood cell maturation [31] (Fig. 2). Furthermore, by examining differentiated erythroid cells, it was found that miR-451 was associated with a decrease in the levels of α-chain, glycophorin-A, and GATA1 transcripts, all of which are crucial for erythropoiesis [31]. This implies that miR-451 may play a role in stimulating erythroid differentiation and maturation, potentially contributing to the pathophysiology of SCA.

### MicroRNA-340-5p

Reducing the amount of miR-340-5p could offer a promising therapy for SCA by boosting the synthesis of fetal hemoglobin and alleviating the manifestations of the condition [7, 32]. Mnika et al. (2019) demonstrated that reducing the amount of miR-340-5p in peripheral blood samples from SCA patients treated with HU can decrease BCL11A stimulation. High levels of miR-340-5p are associated with increased red blood cell destruction and oxidative stress in patients with SCD [7]. By reducing the activity of this small RNA molecule, it may be feasible to decrease these harmful effects, leading to improved stability and function of red blood cells [24, 33] Fig. 1.

### MicroRNA-151-3p

miR-151-3p is recognized for its impact on SCA, being differentially expressed in CD71 + erythroid cells of SCA patients before and after HU treatment. This underscores its potential role in erythropoiesis in SCA and its regulatory effect on KLF1 expression [6, 7, 24, 34], Fig. 2.

### MicroRNA-32-5p

The therapeutic potential of miR-32-5p in SCA treatment lies in its ability to regulate HbF production. Recent studies on peripheral blood samples from SCA patients treated with HU have investigated the inhibitory role of miR-32-5p on BCL11A [7, 35, 36] Fig. 2.

### MicroRNA-15a

MiR-15a plays a crucial role in HbF production in patients with SCA [26]. In SCA, MYB, an inhibitor of γ-globin gene expression, can reduce HbF synthesis. Increased miR-15a expression leads to reduced MYB levels, which causes loss of the inhibitory effect on γ-globin genes and induces HbF production in early erythroid progenitors. Targeting miR-15a or MYB could be a therapeutic strategy to enhance HbF levels in patients with SCA, potentially improving disease outcomes [37].

The findings of an investigation conducted by Pule et al. (2016) showed that miR-15a expression increased in both Umbilical cord blood CD34 + HSCs and K562 cells of SCD patients after HU treatment, and this increase was positively associated with elevated γ-globin expression [17] Fig. 1.

### MicroRNA-26b

MiR-26b stands out among the miRNAs affected by HU treatment [35, 38]. When scientists block miR-26b, γ-globin expression decreases, indicating that it plays a role in boosting γ-globin by targeting and lowering MYB [17, 24]. Alijani et al. in 2014 studied primary erythroid cells grown outside the body and K562 cells exposed to HU and revealed that miR-26b binds to the 3′-untranslated region of MYB mRNA. This binding leads to decreased MYB expression [17, 39].

As stated above, HU increases the expression of certain miRNAs involved in the treatment of sickle cell disease (SCD). Nevertheless, it also leads to increased expression of certain miRNAs that are not of interest for SCD treatment. This can be viewed as an off-target effect of HU treatment in the present study. To optimize SCD treatment, it is crucial to take advantage of the beneficial effects of HU on relevant miRNAs by employing mimics. However, to reduce the side effects of HU treatment, it is important to inhibit the expression of miRNAs that are not of interest. For this purpose, therapeutic RNA inhibitors, such as small interfering RNA (siRNA) or short hairpin RNA (shRNA), are appropriate. A summary of the effects of HU on these miRNA categories is presented in Table 1.

**Table 1:** miRNAs increased by HU treatment

MicroRNA	Model/ Cell line	Target protein	Chromosomal Location	Functional Outcome	Therapeutic Potential	Ref.
miR-210	CD71 + erythroid cells	BCL11A – KLF1	11p15.5	Suppressing erythropoietin, IL-6, TNF-α and IL-1β	Mimic	[10, 24]
miR-144	CD71 + erythroid cells	NRF2	3q29	Increasing Oxidative Stress and repressing of Hmgn2	Inhibition	[38]
miR-29	CD71 + erythroid cells	Sp1 – BCL11A	1q32.2	Increasing the HbF levels and silencing MYB	Mimic	[10]
miR-486-3p	CD34 + erythroid progenitors	BCL11A	8p11.23	Increasing the HbF levels	Mimic	[25]
miR-16-1	CD71 + erythroid cells	MYB	13q14.3	Increasing HbF levels and silencing MYB	Mimic	[10]
miR-451	mouse embryonic stem cells (mESCs)	GATA-1	17q11.2	Promoting erythroid maturation and homeostasis, Upregulating α- and β-globin genes, reducing platelet adhesion	Mimic	[31]
miR-494	CD71 + erythroid cells		14q32.2	Increasing the HbF levels	Mimic	[10]
miR-130	CD71 + erythroid cells	PGC-1α	16q12.1	Downregulating PGC-1α	Inhibition	[28, 29]
miR-340-5P	PBMC	BCL11A	6p21.1	Reduction of oxidative stress	Mimic	[7]
miR-151-3p	CD71 + erythroid cells	KLF1	8q24.3	Increasing the HbF levels	Mimic	[41, 42]
miR-320	CD71 + erythroid cells	CD71	8p21.3.	Increasing the HbF levels	Mimic	[12, 43]
miR-32-5p	PBMC	BCL11A	9p31.3	Reactivation of γ-globin gene expression	Mimic	[40]
miR-26b	primary erythroid cells and K562 cells	MYB	2q35	Downregulating MYB, reducing BCL11A expression	Mimic	[17, 44]
miR-15a	CD34 + HSCs and K562 cells	MYB	13q14	Downregulating MYB, suppressing KLF-1 and BCL11A	Mimic	[17, 45]

## miRNAs decreased by HU treatment

### MicroRNA-222

Functional evaluation of miR-222 in the context of erythropoiesis has primarily been performed using in vitro erythroid differentiation models, particularly CD34⁺ hematopoietic stem and progenitor cells (HSPCs) isolated from peripheral blood or bone marrow and induced toward erythroid lineage under controlled culture conditions. In some mechanistic studies, erythroleukemia cell lines such as K562 cells have been used as a γ-globin–expressing model to investigate miRNA-mediated modulation of globin gene regulation. These experimental systems allowed investigators to assess changes in γ-globin expression following miRNA overexpression or inhibition. Although hydroxyurea was not always directly co-administered in these mechanistic assays, the findings provide a molecular framework suggesting that miR-222 may influence HbF induction pathways that are also targeted by Hydroxyurea [46].

Clinical expression studies evaluating miR-222 in sickle cell disease were largely conducted using ex vivo peripheral blood samples from SCD patients, including cohorts treated with hydroxyurea. miRNA expression profiling was typically performed using quantitative RT-PCR in circulating reticulocytes, total leukocytes, or enriched erythroid fractions. These observational human studies compared miRNA expression patterns between hydroxyurea responders and non-responders, rather than employing a controlled in vitro drug-exposure model. Therefore, the association between miR-222 expression and hydroxyurea efficacy was inferred from differential expression analyses in patient-based clinical models rather than from a dedicated HU-treated cell-line experiment [47, 48].

### MicroRNA-105-5p

Kröppel-like element 1 (KLF1), a protein, plays an important role in the conversion of fetal hemoglobin to adult hemoglobin (HbA1) during development. By examining the erythroid lineage, it was found that by increasing the expression of miRNA-100 and miRNA-105-5p, KLF1 can be reduced through expression, thereby promoting the maintenance of HbF [49, 50]. The severity of SCA may decrease by preventing the buildup of sickle hemoglobin (HbS) as HbF tiers rise and leukemia decreases. It can also affect erythropoiesis and red blood cell development [51, 52] By adjusting the genes involved in this process, enhancing miRNA-105-5p could improve the production and survival of healthy blood cells, which is crucial for patients with SCA [24]. Boosting miR-105-5p could help reduce these harmful effects by regulating genes involved in responses to oxidative stress and cell cycle progression, ultimately enhancing the overall health of red blood cells [53]. Moreover, the interaction of miR-105-5p with other microRNAs that manage HbF production, such as miR-210 and miR-451, could create a synergistic effect that further increases HbF levels [54]. This multi-target strategy could lead to better treatment outcomes in patients with SCA. Therefore, the potential therapeutic benefit of miR-105-5p offers a promising avenue for the medical treatment of SCA [24] Fig. 2.

### MicroRNA-221 (miR-221)

miR-221 might stimulate the formation of red blood cells, which has also enhanced the quality and lifespan of red blood cells [55]. miR-221 has been implicated in the regulation of γ-globin expression and erythroid differentiation in sickle cell disease (SCD). Functional insights were mainly obtained from in vitro erythroid models, including the K562 erythroleukemia cell line and CD34⁺ hematopoietic stem/progenitor cells differentiated toward the erythroid lineage. These systems demonstrated that modulation of miR-221 affects pathways involved in erythroid maturation and HbF regulation. Since hydroxyurea induces HbF as its principal mechanism of action, miR-221 may participate in the molecular network that influences treatment responsiveness [24].

Clinical studies primarily used ex vivo peripheral blood samples from SCD patients treated with hydroxyurea, with miRNA expression measured by qRT-PCR in erythroid or mononuclear cell fractions. Differences in miR-221 expression between treated and untreated patients suggest that hydroxyurea may indirectly modulate this miRNA through its effects on erythroid proliferation and stress erythropoiesis, supporting its potential role as a biomarker of therapeutic response [48] Fig. 1.

### MicroRNA-223

miR-223 impacts SCA by modulating hemoglobin expression and affecting clinical severity [25]. It is found at elevated levels in sickle cells compared to those in normal erythrocytes. MiR-223 directly targets LMO2 mRNA and regulates its expression. Studies in hematopoietic cells have shown that LMO2 is a transcription factor involved in hematopoiesis, and its inhibition affects HbF expression [24]. This interaction leads to decreased LMO2 levels, which affects erythropoiesis and myeloid differentiation. In SCA, miR-223’s modulation of LMO2 prevents HbF production and, along with its role in the immune response and inflammation, aggravates SCA symptoms [7, 10, 34, 56, 57] Fig. 2.

### MicroRNA-96

Normally, miR-96 suppresses γ-globin by binding its mRNA and lowering HbF. Kargutkar et al. (2023) studied CD71 + erythroid cells from SCD patients and healthy individuals. They found that HU treatment reduced miR-96, relieving suppression of gamma-globin mRNA and increasing HbF [10]. Compared to cord blood, adults have higher miR-96 in reticulocytes, which helps silence HbF. Erythroid progenitors with extra miR-96 had a 50% drop in HBG mRNA. Reporter assays confirmed miR-96 directly inhibits HBG [58]. Transfecting K562 cells with anti-miR-96 raised γ-globin 3.54-fold [10].

As mentioned before, HU therapy in sickle cell disease effectively induces fetal hemoglobin (HbF); however, its downregulation of specific microRNAs presents a dual-edged effect. Among the decreased miRNAs, some exert negative regulatory roles, making their suppression beneficial; however, others play positive roles in processes such as HbF induction. This outcome represents a negative, off-target effect of HU that could diminish its overall therapeutic efficacy. To refine and optimize treatment, this negative facet must be counteracted using combinatorial drug strategies. Specifically, the concurrent use of miRNA mimics to restore the beneficial miRNAs lost during HU therapy could overcome this limitation and enhance HU’s therapeutic effect.

Therefore, the primary objective of this review is to propose a framework for refining HU therapy by addressing its complex miRNA-mediated effects. As detailed in Table 2, we summarize the key miRNAs downregulated by HU and propose complementary pharmacological interventions for each, specifying whether a targeted strategy should aim to further inhibit or, conversely, restore their levels using RNA-based therapeutics (e.g., siRNAs, antagomirs, and miRNA mimics). This approach aims to systematically counteract off-target effects and amplify therapeutic synergies, paving the way for more precise and effective combination regimens for sickle cell disease.

**Table 2:** miRNAs decreased by HU treatment

MicroRNA	Model / Cell line	Target protein	Chromosomal Location	Functional Outcome	Therapeutic Potential	Ref.
miR-96	CD71^+^ erythroid cells	γ globin (CDS region)	7q32.2	Reducing HbF levels	Inhibition	[10]
miR-221/-222	Erythroid differentiation models	KLFD	Xp11.3	Reducing HbF levels, modulating signaling pathways like NF-κB and ROS production, suppressing the expression of kit ligand (KL) and c-Kit receptor	Inhibition	[59]
miR-105-5p	PBMC	MYB	7q32.2	Downregulating of MYB, Increasing the fetal hemoglobin (HbF) expression	Mimic	[7]
miR-223	CD71 + erythroid cells	LMO2	Xq12	Reducing IL-6, TNF-α, IL-1β	Mimic	[10]

## circRNA modulation in sickle cell disease and HU therapy

Although circRNAs were discovered several decades ago, there has been recent interest in their ability to interact with RNA-binding proteins and to regulate gene expression. The study of the role of circRNAs in the γ-to-β switch could provide new therapeutic approaches for hemoglobinopathies, such as SCD and β-thalassemia [26].

Recent circRNA studies have significant implications for the study of SCD. Yang et al. (2022) identified the hsa-circRNA-100,466/miR-19b-3p/SOX6 pathway regulating HbF levels [60]. while Chen et al. (2022) showed that circ-0008102 correlates with high HbF and γ-globin expression [61]. Habara (2025) showed that circALS2 is developmentally regulated during erythropoiesis, with different patterns in fetuses and adults [62]. Replication stress induced by HU may affect circRNA biogenesis and splicing, thereby influencing the cicRNA/miRNA pathways that regulate HbF expression. Moreover, circ-0008102 has the potential to be a predictive biomarker for identifying patients who are likely to respond favorably to HU treatment. Table 3 summarizes previous studies that have investigated the role of circRNAs in the molecular mechanisms underlying the pathogenesis of SCD.

Although there is no direct evidence in patients with SCD, the results provide a rationale for future studies to investigate circRNA profiles as biomarkers of therapeutic response and targets for improving HbF induction [62].

**Table 3:** Summary of key studies on circRNAs in hemoglobinopathies and erythropoiesis

Disease/Condition	Sample Type	Key circRNA(s)	Expression Pattern	Molecular Mechanism	Clinical Correlation	Conclusion	Ref
Erythropoiesis (Fetal liver vs. Adult bone marrow)	Erythroblasts from fetal liver and adult bone marrow	circALS2 (upregulated in bone marrow) + 10 other circRNAs	Developmentally regulated; distinct expression patterns between fetal and adult erythroblasts	Distinct interaction networks involving circRNAs, miRNAs, and RNA-binding proteins identified between fetal and adult sources	Associated with developmental stages of erythropoiesis	Suggests a novel regulatory circuit in human erythropoiesis; candidates need experimental validation for therapeutic potential	[62]
β-thalassemia carriers with elevated HbF	Peripheral blood from β-thalassemia carriers vs. healthy individuals	hsa-circRNA-100,466 (downregulated)	Downregulated in carriers with high HbF; strong negative correlation with HbF and HbA2 levels	ceRNA pathway: hsa-circRNA-100,466/miR-19b-3p/SOX6; validated by qRT-PCR, RIP, and dual-luciferase assays	Negatively correlated with HbF and HbA2 levels	Reveals a novel regulatory pathway for HbF induction and suggests potential therapeutic targets for β-thalassemia	[60]
Pediatric β-thalassemia (β-thal)	Peripheral blood from pediatric patients (HbF ≥ 5.0%, HbF < 5.0%, and healthy controls)	circ-0008102 (upregulated in high HbF group)	Downregulated in carriers with high HbF; strong negative correlation with HbF and HbA2 levels	Putative miRNA sponge (top candidates: miR-372-3p, miR-329-5p, miR-198, miR-152-5p, miR-627-3p); involved in Th17 differentiation and stem cell pluripotency pathways	Positively correlated with HbF, GGT, β-globin mRNA, **and** γ-globin mRNA; negatively correlated with MCV, MCH, HbA, and Cr	Preliminary evidence that circ-0008102 is an effective biomarker for pediatric β-thal with high HbF; participates in pathogenesis via γ-globin regulation	[61]

## Discussion

Our results shed light on the complex relationship between HU efficacy and the regulation of individual miRNAs in SCD, and this molecular basis underlies the heterogeneity of responses. Although this study serves to validate the need for the development of miRNA-targeted therapies, either as adjuncts to HU or as standalone agents, the translation of this work from the laboratory bench to the bedside is dependent on overcoming a number of complex challenges. This discussion will consider these challenges in turn, focusing on the areas of delivery, specificity, and safety, before placing these in the context of the realities of implementation in RLS, where the vast majority of patients with SCD are found [24, 63].

## Biological and engineering barriers to miRNA delivery in SCD

The therapeutic concept of restoring beneficial miRNAs (e.g., using mimics of miR-451 and miR-486-3p to promote healthy erythropoiesis) or inhibiting detrimental ones (e.g., using antagomirs against miR-144 and miR-494 to suppress fetal hemoglobin inhibitors) is elegant in principle [64, 65]. However, the systemic delivery of these synthetic oligonucleotides to their intended target, hematopoietic stem and progenitor cells (HSPCs) within the bone marrow, remains a formidable challenge.

Oligonucleotides are prone to degradation by nucleases in the bloodstream and require advanced delivery systems for their effective delivery. Although lipid nanoparticles (LNPs) have been historically successful with mRNA vaccines, their use in SCD is not straightforward [66, 67]. The natural biodistribution of LNPs is preferential for the liver, which is not ideal for targeting HSPCs and also raises concerns for hepatotoxicity, which is a major concern in SCD patients who may already have iron overload-induced liver disease [68, 69]. Furthermore, repeated administration, as would be required for a chronic condition such as SCD, could trigger immunogenic responses against LNP components, diminishing efficacy and causing adverse effects [67, 69]. Viral vectors, such as adeno-associated viruses (AAV), have high transduction efficiency but pose unacceptable risks for non-malignant diseases, including insertional mutagenesis, oncogenic activation, and strong immunogenicity. The emergence of non-viral synthetic nanoparticles provides a more promising approach [70, 71]. These carriers can be engineered with surface ligands (e.g., antibodies or aptamers against CD34 or CD117) to actively target HSPCs [72]. However, scaling the manufacturing of such complex, targeted systems to good manufacturing practice (GMP) standards is a major hurdle [73]. Providing stability during storage and administration and preventing premature release of the miRNA payload are other pharmacokinetic challenges that remain to be solved. Without a reliable, safe, and highly specific delivery system, the therapeutic value of miRNA modulation, such as the anti-fibrotic miR-29 family or the apoptosis-regulating miR-15/miR-16-1 cluster, cannot be fully exploited [73, 74].

## The challenge of off-target effects and therapeutic specificity

A fundamental aspect of miRNA biology is that a single miRNA can bind to the 3’ untranslated region (3’ UTR) of hundreds of different messenger RNAs (mRNAs), creating a complex regulatory network [75, 76]. This pleiotropy is a double-edged sword. While it allows for the coordinated regulation of entire biological pathways, it also means that a therapeutic mimic or inhibitor will inevitably cause unintended, off-target effects [68, 77–79]. For instance, mimics of miR-210, a master regulator of the hypoxic response, could improve certain aspects of SCD pathophysiology but may also have profound off-target effects on angiogenesis and cell proliferation in non-hematopoietic tissues [80]. Similarly, inhibiting miR-221/-222 to enhance c-KIT expression and erythropoiesis could inadvertently impact endothelial cell function and tumor suppression pathways where these miRNAs are also active [81, 82]. The off-target risk is mainly due to the “seed sequence” (nt 2–8) of the miRNA, which may display partial complementarity to thousands of transcripts. Although computational predictions can identify some of these interactions, their accuracy is low, and only extensive empirical validation by transcriptomics and proteomics analyses can uncover the full extent of off-target gene regulation. To counter these issues, complex chemical modifications of the oligonucleotide backbone would be necessary to improve target binding affinity, and extensive dose-finding studies would be needed to reduce off-target interactions, and perhaps the creation of “suicide gene” safety switches for delivery vehicles. The use of broadly active miRNAs such as miR-320, miR-223, miR-26b, miR-96, miR-130, miR-105-5p, miR-340-5P, miR-148-3p, miR-151-3p, miR-32-5p, and miR-199a-5P would require an unusually high level of diligence to ensure that the benefits of on-target effects are not negated by the liabilities of off-target effects [83–86].

## Safety, immunogenicity, and the narrow therapeutic window

In addition to off-target gene expression, miRNA therapeutics have direct safety concerns. The immune system is designed to recognize foreign nucleic acids, and the use of synthetic RNA oligonucleotides can activate the recognition receptors, including Toll-like receptors (TLR7 and TLR8) and RIG-I. This initiates an intense inflammatory response, with the secretion of type I interferons and pro-inflammatory cytokines [87–90]. In SCD, a disease already characterized by chronic inflammation, such immune activation could be catastrophic, potentially precipitating a vaso-occlusive crisis [91–93]. Moreover, saturating the endogenous RNA-induced silencing complex (RISC) with high doses of a therapeutic mimic can competitively displace endogenous miRNAs, disrupting normal cellular homeostasis and leading to dose-dependent cytotoxicity [83, 84] This establishes a therapeutic index that could be dangerously small. Establishing a dose that is adequate to modulate the target transcript in HSPCs, yet not induce systemic inflammation or cellular toxicity, will be a complex task that will require extensive preclinical safety modeling and phase I clinical studies [94]. The safety profile is paramount and must be evaluated in the specific context of SCD pathophysiology, in which endothelial dysfunction, immune dysregulation, and organ damage are pre-existing conditions.

## Overcoming implementation barriers in resource-limited settings

Although the aforementioned scientific challenges are of a global nature, they also satisfy a substantial list of socioeconomic challenges that are most evident in the RLS of sub-Saharan Africa and India, where the SCD burden is also the highest. Notably, the current standard of care, HU, faces substantial challenges in this regard. Though it is cost-effective (less than $1 per day), this is still not possible for many families, and the required laboratory infrastructure (such as complete blood counts) is not available [3, 95, 96]. In this context, the idea of developing and administering a highly effective miRNA therapy, which is likely to cost thousands of dollars per treatment, appears to be improbable. The infrastructure needed for administering such therapies, such as a cold chain for storage, infusion centers, and sophisticated molecular labs for monitoring the efficacy and toxicity of the treatment, is far beyond the current healthcare infrastructure available in most RLS. This is a major ethical issue: the development of a highly effective but unaffordable treatment could potentially worsen the disparity in global health, leading to a two-tiered system of healthcare in which life-altering treatments are only available to patients in developed nations [97].

## Conclusion

In light of the above, the current research on the involvement of miRNAs and circRNAs in HU-induced HbF expression has great potential to improve our knowledge of SCA therapy. Future research will be able to achieve a new level of understanding of HU efficacy by focusing more on these molecular processes, which will help develop a therapy. Further research is required to discover new miRNAs and circRNAs affected by HU, which will uncover new regulatory pathways. Finally, by utilizing our current knowledge of these molecular processes, we will be able to develop future therapies that will help optimize HbF production and improve SCA patient outcomes.

## Data Availability

Not applicable.
